# Pelvic Floor Disorders in Women with Breast Cancer and Gynaecological Cancer: Real-World Data from a Prospective Multicentre Cross-Sectional Survey

**DOI:** 10.3390/jcm15166218

**Published:** 2026-08-11

**Authors:** Carolin Schröder, Sheila Bonilla Moran, Verena Kirn, Andree Faridi, Philipp Meyer-Wilmes, Laila Najjari, Fabinshy Thangarajah, Sebastian Hentsch, Christian Kurbacher, Laura Tascón Padrón, Lucia A. Otten, Eva K. Egger, Alexander Mustea, Dominique Koensgen

**Affiliations:** 1 Department of Gynaecology and Gynaecological Oncology, University Hospital Bonn, University of Bonn, 53127 Bonn, Germany; 2 Department of Senology, University Hospital Bonn, University of Bonn, 53127 Bonn, Germany; 3 Department of Gynaecology and Obstetrics, University Hospital RWTH Aachen, Pauwelsstrasse 30, 52074 Aachen, Germany; 4 Department of Gynaecology and Obstetrics, University Hospital Essen, Hufelandstraße 55, 45147 Essen, Germany; 5 Department of Gynaecology and Obstetrics, University Hospital Düsseldorf, Moorenstraße 5, 40225 Düsseldorf, Germany; 6 Gynaecologic Centre Bonn-Friedensplatz, Department of Gynaecology I (Gynaecologic Oncology), 53111 Bonn, Germany

**Keywords:** pelvic floor disorder, gynaecological cancer, breast cancer, quality of life

## Abstract

**Background/Objectives:** Women with breast cancer (BC) and gynaecological cancer (GC) often undergo extensive oncological treatment, which can lead to pelvic floor disorders (PFD). However, there is still limited evidence regarding their real-world treatment practices, patient counselling, and prevalence. This study aims to systematically evaluate the prevalence of PFD and current treatment approaches, identify unmet needs, and improve future care. **Methods:** This observational multicentre survey was conducted at four university hospitals and one gynaecological oncology outpatient clinic in Germany. Women with BC and GC, who had undergone at least one oncological treatment, completed anonymously a study-specific questionnaire and the validated German Pelvic Floor Questionnaire. Recruitment was undertaken over a period of nine months, reflecting real-world daily care. **Results:** A total of 246 patients were included (median age 57 years, range 30–88); 74% were postmenopausal, and 52% had at least one vaginal delivery. BC was the most common diagnosis (63.8%). The most common symptoms were urinary incontinence (22.3% in BC, 38.1% in GC) and urgency (32.5% in BC, 35.7% in GC). Pelvic radiotherapy was significantly associated with increased urgency symptoms (*p* = 0.017), pelvic surgery with urgency symptoms (*p* = 0.048) and dyspareunia (*p* = 0.045), and antihormonal therapy with vaginal dryness (*p* = 0.028). The combination of chemotherapy and antihormonal therapy was associated with significantly higher rates of vaginal dryness in women with GC, whereas no such association was observed in women with BC (*p* = 0.036). Among the 108 patients classified as having new-onset symptoms, 51 (47.2%) reported symptom onset within one year of the cancer diagnosis. Overall, 34.1% of patients reported impairment in more than one patient-reported domain, including daily life, social life, sexual well-being, and psychological well-being. The median study-specific impact rating for the most bothersome pelvic floor symptom was 5/10. Only 28.0% of patients received prior counselling on PFD, and 19.9% were actively asked about symptoms. In total, 26.0% received urogynaecological treatment, whereas 15.4% reported unmet treatment needs. **Conclusions:** PFD is highly prevalent in women with BC and GC. Symptoms are clinically relevant but underestimated in routine care. Despite the availability of effective treatment options, significant gaps exist in patient counselling, early detection, and access to specialised care.

## 1. Introduction

In 2020, approximately 96,710 women in Germany were diagnosed with breast (BC) and gynaecological cancers (GC, including cancers of the uterus, cervix, ovaries, vulva, and vagina) [1]. Pelvic floor disorders (PFD), including urinary and faecal incontinence as well as pelvic organ prolapse, affect up to 46% of adult women worldwide and represent a major burden on quality of life (QoL), sexual health, and daily functioning [2]. Women with breast and gynaecological cancers may be particularly vulnerable to developing PFD due to the effects of oncological treatments such as surgery, radiotherapy, chemotherapy, and antihormonal therapy. Advances in early detection and multimodal therapies have significantly improved survival rates, resulting in a growing population of long-term survivors. Systematic reviews highlight the high prevalence of these treatment side effects: dyspareunia affects nearly 45–60% of women treated for cervical cancer and about 40% of those after endometrial cancer treatment; urinary and faecal incontinence are reported by almost 40% of patients in both groups [3,4,5]. Among ovarian cancer survivors, sexual health can be affected in up to 80% [5,6]. Beyond oncological treatment, additional risk factors include previous childbirth, age, and menopausal status. Importantly, conservative interventions such as pelvic floor muscle training, local estrogen therapy, vaginal dilatators, and patient education have been shown to reduce symptoms and significantly improve sexual health and QoL [3,7,8,9]. Nevertheless, evidence about real-world practice, patient counselling, and the treatment of PFD in patients with breast and gynaecological cancer remains limited.

The primary aim of this study was to evaluate real-world counselling and treatment practices for patients with PFD and with breast and gynaecological malignancies. Secondary objectives included a hypothesis-generating assessment of the prevalence of PFD among patients with gynaecological malignancies to identify unmet needs in current clinical care.

## 2. Methods and Material

The study is a multicentre, anonymised, cross-sectional observational survey with prospective recruitment and data collection to assess the real-world prevalence and management of PFD among women diagnosed with BC and GC. The study did not include any intervention and was only an observational study. It was coordinated and conducted by the Department of Gynaecology and Gynaecological Oncology at the University Hospital Bonn. Overall, four university hospitals in Germany and one outpatient gynaecological oncology clinic recruited patients into the study between the 1 April and the 31 December 2025.

Patients were recruited by convenience sampling in outpatient clinics, inpatient wards, and during follow-up visits. BC and GC patients were recruited using the same procedure: patients were approached at the participating centres and asked whether they wished to participate anonymously in the survey. BC and GC were investigated together because both patient groups may experience pelvic floor, sexual, and vulvovaginal symptoms following oncological treatment, although the underlying mechanisms may differ. As uniform screening logs were not maintained across the participating centres, the exact numbers of eligible patients approached and patients who declined participation could not be reconstructed retrospectively. The primary aim of the study was to provide a comprehensive real-world overview of the current prevalence of PFD and related treatment practices. This included the prevalence of urinary and faecal incontinence, pelvic organ prolapse symptoms, and the way patients were counselled and treated. Women aged at least 18 years with a confirmed diagnosis of BC or GC (including cancers of the uterus, cervix, ovaries, vulva, and vagina) who had received at least one oncological therapy, such as surgery, chemotherapy, radiotherapy, or antihormonal therapy, were invited to participate in this study. Data were collected anonymously using two questionnaires.

The study-specific questionnaire (SSQ) was developed for this study (Supplementary File S1). It included 33 questions divided into three sections: A: demographic and clinical data (such as age, menopausal status, body mass index, type of cancer and detailed treatment history); B: pelvic floor symptoms (such as urinary and faecal incontinence, pelvic organ prolapse, sexual disorder, and vaginal dryness, their impact on daily activities, social life, sexual well-being and psychological well-being); C: patient-reported counselling (including information received prior to treatment, perceived quality of care, and any therapeutic interventions undertaken, ranging from physiotherapy to medication or surgical procedures). In Part B, participants were asked to identify their most bothersome current pelvic floor symptom and rate its impact on daily life using a study-specific 0–10 numerical rating scale, with 0 indicating no impairment and 10 indicating very severe impairment. This single-item rating assessed the impact of the most bothersome symptom and was not designed or validated as a measure of overall PFD burden or general quality of life. The effects of pelvic floor symptoms on daily life, social contacts, sexual well-being, and psychological health were assessed independently using separate items. Response options were “yes” and “no” for each domain, with “not sexually active” provided as an additional response option for sexual well-being. Participants selecting “not sexually active” were not classified as having no impairment in this domain.

In addition to the SSQ, participants completed the validated German Pelvic Floor Questionnaire (GPFQ), which evaluates four specific domains: bladder function, bowel function, pelvic organ prolapse, and sexual function [10]. Each GPFQ domain score is standardised to a range from 0 to 10, and the four domain scores are summed to obtain a total score ranging from 0 to 40. Higher scores indicate greater symptom severity and pelvic floor dysfunction. Both questionnaires were filled out once and were fully anonymised.

The statistical analysis was conducted using the statistical software SPSS 29.0.2.0 (IBM Corp., released 2023, IBM SPSS Statistics for Windows, Version 29.0, Armonk, NY, USA: IBM Corp). Descriptive statistics were used to summarise patient characteristics and symptom prevalence (means with standard deviations, medians with ranges, and frequencies with percentages). Associations between categorical variables were analysed using Pearson’s chi-square test or Fisher’s exact test, as appropriate. For continuous or ordinal questionnaire outcomes, non-parametric comparisons were performed using the Mann–Whitney U test, with a significance level set at *p* ≤ 0.05. Given the exploratory and hypothesis-generating nature of the study, no adjustment for multiple testing was performed. No questionnaires were excluded because of missing data. Missing responses to individual items were treated as missing, and each analysis was based on the available responses for the respective variable.

The study was approved by the Ethics Committee of the University of Bonn on 25 February 2025 (reference no. 2025-10-BO) prior to recruitment of patients. Participants were recruited from April to December 2025. Since this was an observational study with no intervention, the study was retrospectively registered in the German Clinical Trials Register on 23 December 2025 (DRKS00038814) after recruitment had begun but before it was completed. The delayed registration was due to administrative reasons. The study was conducted in accordance with the protocol approved by the ethics committee, and the retrospective registration did not affect patient management, recruitment procedures, data collection, statistical analysis or outcome assessment.

## 3. Results

A total of 246 patients completed the survey and were included, with a median age of 57 years and a median BMI of 25 kg/m^2^. The majority of patients were postmenopausal (74%) and had experienced at least one vaginal delivery (52%). In total, 43.9% of patients had at least one comorbidity, with the majority living in a partnership (58.9%). Breast cancer (BC) was the most common diagnosis (63.8%), and most patients had undergone chemotherapy (67.5%), and were under current treatment (81.3%). Table 1 summarises patients’ characteristics for the total cohort and separately for patients with BC and GC.

Distribution of PFD by tumour entity: Urinary incontinence was significantly more frequent among patients with GC than among those with BC. The distribution of symptoms is summarised in Table 2.

Distribution of PFD by oncological treatment: Pelvic radiotherapy was significantly associated with increased urgency symptoms (*p* = 0.017), pelvic surgery with urgency symptoms (*p* = 0.048) and dyspareunia (*p* = 0.045) and antihormonal therapy with vaginal dryness (*p* = 0.028). No other treatment-related differences were statistically significant, although trends were observed (Table 3). In exploratory subgroup analyses, among patients with GC, vaginal dryness was significantly more frequent in women receiving combined chemotherapy and endocrine therapy than in those receiving either treatment modality alone (*p* = 0.036). In contrast, no significant associations were observed among BC patients.

These findings were supported by GPFQ results (Table 4), demonstrating significantly higher bladder scores in patients after pelvic radiotherapy (*p* = 0.009) and higher overall scores in those undergoing pelvic surgery (*p* = 0.040). No other statistically significant associations were observed, although trends were noted for pelvic surgery and selected symptoms.

Patient-reported impact of pelvic floor symptoms: Impairment in only one domain (daily life, social life, sexual well-being or psychological well-being) was reported by 37 patients (15%), whereas 84 patients (34.1%) reported symptoms in more than one domain. Among patients with isolated impairment, sexual well-being was the most frequently affected domain. On the study-specific 0–10 numerical rating scale, the median impact of the most bothersome pelvic floor symptom on daily life was 5, with higher scores indicating greater impairment. Overall, 83 patients (33.8%) reported no impairment in any of the assessed domains (daily activities, social life, sexual well-being and psychological well-being).

Temporal relationship between PFD and oncological treatment: Regarding the temporal course of pelvic floor symptoms, 76 patients (30.9%) reported that symptoms were present before oncological treatment. Of these, 53 patients (69.7%; 21.5% of the total cohort) reported that their symptoms had remained unchanged, whereas 23 patients (30.3%; 9.3% of the total cohort) reported that their symptoms had subsequently worsened.

A further 108 patients (43.9%) reported current symptoms that had not been present before oncological treatment and were therefore classified as having new-onset symptoms. Among these patients, symptom onset was reported within one year of the cancer diagnosis by 51 patients (47.2%), between one and five years by 10 patients (9.3%), and more than five years after diagnosis by 2 patients (1.9%). Information on the timing of symptom onset was missing or not evaluable for 45 of the 108 patients with new-onset symptoms (41.7%). The remaining 62 participants (25.2%) could not be classified because information on symptoms before oncological treatment was unavailable.

Counselling and care pathways: Only 69 patients (28.0%) reported being informed about potential pelvic floor symptoms, while the majority (143 patients, 58.1%) had not received such information. Correspondingly, 78 patients (31.7%) indicated that they would have preferred more information.

Direct questioning about pelvic floor symptoms by healthcare providers was uncommon, with only 49 patients (19.9%) reporting that they had been actively asked. Among patients experiencing symptoms, the main reasons for not seeking earlier help included a lack of knowledge about available treatment options (23 patients, 9.4%) and uncertainty about where to obtain help (13 patients, 5.3%). Additional barriers included personal or psychosocial factors such as embarrassment or not feeling taken seriously.

**Figure 1:**
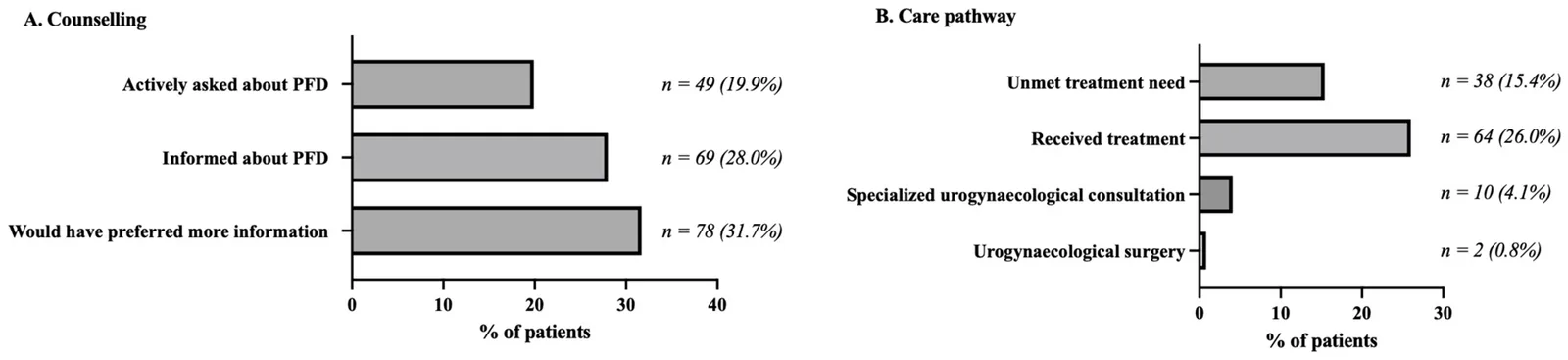
Counselling and care pathway. Percentages were calculated using the total number of patients (*n* = 246).

Overall, 64 patients (26.0%) reported receiving treatment for pelvic floor symptoms, while 38 patients (15.4%) reported no treatment despite experiencing symptoms. Among those who received treatment, satisfaction was moderate: most patients reported neutral (42 patients, 17.1%) or positive (31 patients, 12.6%) experiences, whereas dissatisfaction was relatively rare (9 patients, 3.7%). Only a small proportion of patients (*n* = 10, 4.1%) reported having attended a specialised urogynaecological consultation. Only 2 patients received urogynaecological surgery (0.8%). Figure 1 summarised counselling and care pathways.

## 4. Discussion

This multicentre real-world cross-sectional study provides detailed insights into the prevalence, management, and clinical importance of PFD in women with breast and gynaecological malignancies. The findings indicate that PFD is common in this patient population. Among patients with new-onset symptoms and evaluable timing data, symptom onset was most frequently reported within one year of the cancer diagnosis. Importantly, the results also reveal substantial gaps in patient counselling, symptom recognition, and access to appropriate care.

Given that PFD affect up to 46% of women in the general population, women undergoing treatment for breast and gynaecological malignancies may represent a particularly vulnerable subgroup [2]. Accordingly, the observed prevalence of PFD in our cohort aligns with previous literature reporting high rates of urinary, bowel, and sexual disorders following oncological treatment. Systematic reviews have shown that urinary and faecal incontinence affect up to 40% of women after treatment for cervical and endometrial cancer, while dyspareunia may occur in up to 45–60% of patients [3,4,5]. Similarly, sexual disorder has been reported in up to 80% of ovarian cancer survivors [6]. Our findings confirm that PFD are highly common across tumour types; however, unlike previous reports, tumour type was not significantly associated with most pelvic floor symptoms. These findings suggest that treatment-related factors warrant further investigation, although confounding by baseline differences between the tumour groups cannot be excluded. Exploratory analyses identified several associations between oncological treatment modalities and PFD. Pelvic radiotherapy was associated with a higher prevalence of urgency symptoms, whereas antihormonal therapy was associated with vaginal dryness. Among women with GC, combined chemotherapy and endocrine therapy were associated with a higher prevalence of vaginal dryness than either treatment modality alone; no corresponding association was observed among patients with BC. However, this finding was limited to the symptom-based analysis and was not reflected in the GPFQ domain or total scores. The GPFQ analyses demonstrated higher bladder scores among patients who had undergone pelvic radiotherapy and higher total scores among those who had undergone pelvic surgery. These findings are consistent with evidence that radiotherapy can cause long-term changes in bladder function and pelvic tissues and that endocrine therapies may adversely affect vaginal and sexual health [7,8]. However, most treatment-related associations did not reach statistical significance, likely because of the limited subgroup sizes. These exploratory findings should therefore be interpreted cautiously and require confirmation in larger studies.

Beyond symptom prevalence, our study offers important insights into real-world care pathways. Despite the high burden of PFD, most patients (58%) reported not being informed about potential side effects of oncological treatment, which is even higher than the 34% in another cross-sectional, questionnaire-based monocentre study who stated they received no information on urogynaecological symptoms caused by oncological treatment. [11]. Furthermore, only a minority of patients were actively asked about symptoms by healthcare providers, and only a small proportion received specialised urogynaecological care. These findings are especially striking given that effective conservative interventions, including pelvic floor muscle training, local estrogen therapy, and structured patient education, have been shown to significantly improve symptoms and quality of life [3,9,12]. In our study, under 1% of patients underwent urogynaecological surgery. Other studies report concomitant oncological and reconstructive-urogynaecological surgeries, which are feasible but associated with a low but statistically significant increase in adverse events [13,14]. Overall, the discrepancy between available interventions and their limited use in clinical practice indicates a substantial unmet need.

Patient-reported barriers further illustrate this unmet need. Lack of knowledge about treatment options and uncertainty about where to obtain appropriate care were the most frequently reported barriers, indicating a need to improve both patient education and healthcare system structures. Psychosocial factors, including embarrassment and feeling inadequately supported, were also reported. These findings emphasise the importance of proactive communication and systematic symptom assessment during routine oncological care and follow-up. Greater awareness of PFD among healthcare providers and patients may facilitate earlier symptom recognition, timely referral to specialised care, and better integration of urogynaecological services into supportive oncological care.

The strengths of this study include its multicentre design, prospective recruitment, and a relatively large real-world cohort drawn from four university hospitals and one outpatient gynaecological oncology clinic. The inclusion of patients with both breast and gynaecological malignancies, together with a broad range of treatment exposures, enabled an exploratory assessment of pelvic floor symptoms across different oncological care settings and patient groups. The combination of the validated GPFQ with the SSQ allowed standardised assessment of pelvic floor symptoms while capturing clinically relevant aspects not covered in detail by the GPFQ, including patient counselling, perceived information needs, treatment uptake, barriers to seeking care, and care pathways. Furthermore, anonymous data collection may have facilitated the reporting of sensitive symptoms related to urinary, bowel, and sexual health.

However, several limitations should be recognised. First, the cross-sectional design precludes causal inference and does not allow assessment of symptom progression over time. Pelvic floor status before oncological treatment was assessed retrospectively by patient report rather than prospectively, making the findings susceptible to recall bias and reporting inaccuracies. Second, although the validated GPFQ was used, the additional SSQ was not independently validated. Third, pelvic floor symptoms were assessed exclusively using patient-reported data, without confirmation by an objective clinical or urogynaecological examination. Fourth, uniform screening logs were not maintained across the participating centres. The number of eligible patients approached and those who declined participation could therefore not be reconstructed, precluding calculation of a response rate. Selection bias cannot be excluded, as participation may have differed according to pelvic floor symptom severity, disease burden, or treatment burden. Fifth, missing responses to individual items resulted in variable-specific sample sizes. Furthermore, the analyses were not adjusted for potential confounders using multivariable methods. Differences in pelvic floor symptoms could therefore not be attributed solely to oncological treatment, but rather to age, parity, menopausal status, comorbidities, or other differences between the tumour groups. Multiple statistical tests were performed without correction because of the exploratory nature of the study, increasing the risk of type I error. Finally, the relatively small subgroup sizes limited statistical power, particularly for comparisons between individual treatment modalities.

This study should be viewed as an exploratory and hypothesis-generating pilot study and a basis for future prospective research. A longitudinal study design with repeated assessments using validated questionnaires at different time points—from initial diagnosis through treatment and follow-up—would provide a more detailed understanding of the development and progression of PFD in this patient population and stratified by tumour entity. Such an approach would help identify critical intervention periods, improve risk stratification, and support the implementation of targeted preventive and therapeutic strategies.

## 5. Conclusions

PFD is common and clinically significant among women with breast and gynaecological cancers, yet it remains inadequately addressed in current clinical practice. Enhanced patient counselling, systematic symptom assessment, and better integration of urogynaecological care into oncological pathways are urgently needed. Future prospective studies are essential to further elucidate symptom trajectories and optimise patient-centred care.

## Figures and Tables

**Table 1:** Patients’ characteristics.

	Total Cohort *n* = 246	Breast Cancer *n* = 157	Gynecological Cancer *n* = 84
Age median (range), years	57 (30–88)	56 (30–88)	58 (31–87)
BMI median (range), kg/m^2^	25 (15–47)	25 (15–47)	25 (16–45)
Menopausal status, *n* (%)			
Premenopausal	20 (8.1)	11 (7)	8 (9.5)
Perimenopausal	22 (8.9)	15 (9.6)	7 (8.3)
Postmenopausal	182 (74)	115 (73.2)	63 (75.1)
Missing	22 (8.9)	16 (10.2)	6 (7.1)
Parity, *n* (%)			
≥1 vaginal delivery	128 (52)	78 (49.7)	49 (58.4)
≥1 vacuum extraction	30 (12.2)	21 (13.3)	7 (8.3)
No vaginal delivery	116 (47.2)	78 (49.7)	34 (40.5)
≥1 caesarean section	51 (20.7)	35 (22.2)	16 (19.1)
Missing	2 (0.8)	1 (0.6)	1 (1.2)
Comorbidities, n (%)			
≥1 comorbidity	108 (43.9)	66 (42)	39 (46.4)
No comorbidities	95 (38.6)	65 (41.4)	30 (35.7)
Missing	43 (17.5)	26 (16.6)	15 (17.9)
Smoking status, *n* (%)			
Current smoker	29 (11.8)	15 (9.6)	13 (15.5)
No smoker	215 (87.4)	142 (90.4)	69 (82.1)
Missing	2 (0.8)	0	2 (2.4)
Marital status *n* (%)			
Married/in partnership	145 (58.9)	82 (52.2)	59 (70.2)
Single	61 (24.8)	50 (31.8)	10 (11.9)
Divorced/separated	16 (6.5)	10 (6.4)	6 (7.2)
Widowed	23 (9.3)	15 (9.6)	8 (9.5)
Missing	1 (0.4)	0	1 (1.2)
Educational level			
No/Low formal education	3 (1.2)	1 (0.6)	2 (2.4)
Secondary education	156 (63.4)	96 (61.2)	58 (69)
Higher education (university degree)	86 (35.0)	59 (37.6)	24 (28.6)
Missing	1 (0.4)	1 (0.6)	0
Diagnosis, *n* (%)			
Breast cancer	157 (63.8)		
Ovarian cancer	40 (16.3)		
Endometrial cancer	20 (8.1)		
Vulvar cancer	12 (4.9)		
Cervical cancer	11 (4.5)		
Other malignancy	1 (0.4)		
Missing	5 (2)		
Years since cancer diagnosis, *n* (%)	*n* = 238 (97)	*n* = 149 (95)	*n* = 84 (100)
median (range)	2 (1–46)	3 (1–46)	2 (1–31)
Previous treatment modalities, *n* (%)			
Surgical treatment	138 (56.1)	94 (59.9)	40 (47.6)
Pelvic surgery	52 (21.1)	34 (21.7) ^a^	14 (16.7) ^a^
Chemotherapy	166 (67.5)	101 (64.3)	61 (72.6)
Radiotherapy	110 (44.7)	70 (44.6)	39 (46.4)
Pelvic radiotherapy	25 (10.2)	16 (10.2) ^a^	8 (9.5) ^a^
Antihormonal therapy	75 (30.5)	49 (31.2)	26 (31)
Oncological treatment status, *n* (%)			
Currently under treatment	200 (81.3)	119 (75.8)	78 (92.9)
Follow-up/no active treatment	36 (14.6)	31 (19.7)	4 (4.8)
Missing	10 (4.1)	7 (4.5)	2 (2.4)

Percentages were calculated using the total number of patients in the respective column as the denominator, with missing responses reported as a separate category where applicable. For variables allowing multiple responses, categories were not mutually exclusive, and percentages may therefore sum to more than 100%. Five patients with missing tumour classification were included in the total cohort but excluded from the tumour-specific columns. BMI, body mass index. (^a^) Pelvic surgery and radiotherapy were based on patient-reported surgical history and were not necessarily related to the current malignancy. The indication and timing of these procedures were not captured in sufficient detail. It may reflect a history of more than one malignancy or a different interpretation of the questionnaire item. Owing to the anonymous study design, the indication for surgery and potential misunderstandings could not be verified retrospectively.

**Table 2:** Distribution of pelvic floor disorders by tumour entity.

Symptom *n* (%)	Breast Cancer (*n* = 157)	Gynaecological Cancer (*n* = 84)	*p*-Value
Urinary incontinence	35 (22.3)	32 (38.1)	0.009
Urgency	51 (32.5)	30 (35.7)	0.613
Prolapse symptoms	6 (3.8)	2 (2.4)	0.717 *
Dyspareunia	14 (8.9)	12 (14.3)	0.201
Vaginal dryness	28 (17.8)	22 (26.2)	0.127

Data are presented as *n* (%). Patients could report more than one pelvic floor symptom; therefore, symptom categories were not mutually exclusive, and percentages within each tumour group may sum to more than 100%. Percentages were calculated using the total number of patients in the respective tumour group as the denominator. Dyspareunia percentages were calculated among all patients in the respective tumour group, irrespective of current sexual activity, and may therefore underestimate its prevalence among sexually active patients. Five patients with missing tumour classification were excluded from the tumour-group comparison. Pearson’s chi-squared test was used, except for *, for which Fisher’s exact test was applied because of small expected cell counts.

**Table 3:** Distribution of pelvic floor disorders by oncological treatment.

Symptom Category *n* (%)	Chemotherapy Yes: *n* = 166; No: *n* = 80	Antihormonal Therapy Yes: *n* = 75; No: *n* = 171	Pelvic Radiotherapy Yes: *n* = 25; No: *n* = 221	Pelvic Surgery Yes: *n* = 52; No: *n* = 194
Urinary incontinence	Yes: 50 (30.1)	Yes: 23 (30.7)	Yes: 11 (44)	Yes: 19 (36.5)
	No: 22 (27.5)	No: 49 (28.7)	No: 61 (27.6)	No: 53 (27.3)
	*p* = 0.672	*p* = 0.750	*p* = 0.088	*p* = 0.194
Urgency symptoms	Yes: 54 (32.5)	Yes: 26 (34.7)	Yes: 14 (56)	Yes: 24 (46.2)
	No: 31 (38.8)	No: 59 (34.5)	No: 71 (32.1)	No: 61 (31.4)
	*p* = 0.337	*p* = 0.980	*p* = 0.017	*p* = 0.048
Prolapse symptoms	Yes: 6 (3.6)	Yes: 5 (6.7)	Yes: 1 (4.0)	Yes: 2 (3.8)
	No: 2 (2.5)	No: 3 (1.8)	No: 7 (3.2)	No: 6 (3.1)
	*p* = 1.000 *	*p* = 0.059 *	*p* = 0.581 *	*p* = 0.677 *
Dyspareunia	Yes: 21 (12.7)	Yes: 7 (9.3)	Yes: 1 (4.0)	Yes: 10 (19.2)
	No: 7 (8.8)	No: 21 (12.3)	No: 27 (12.2)	No: 18 (9.3)
	*p* = 0.367	*p* = 0.503	*p* = 0.327*	*p* = 0.045
Vaginal dryness	Yes: 40 (24.1)	Yes: 22 (29.3)	Yes: 4 (16.0)	Yes: 13 (25.0)
	No: 11 (13.8)	No: 29 (17.0)	No: 47 (21.3)	No: 38 (19.6)
	*p* = 0.061	*p* = 0.028	*p* = 0.538	*p* = 0.393

Data are presented as *n* (%). For each treatment modality, percentages were calculated separately for patients who received the treatment (“yes”) and those who did not (“no”), using the group sizes shown in the column headings as denominators. Symptom categories were not mutually exclusive, and patients may have received more than one treatment modality. Dyspareunia percentages were calculated irrespective of current sexual activity and may therefore underestimate its prevalence among sexually active patients. For each treatment modality, the *p*-value compares the “yes” and “no” groups. Pearson’s chi-squared test was used, except for comparisons marked with an asterisk (*), for which Fisher’s exact test was applied because of small expected cell counts.

**Table 4:** Association between treatment modalities and the German pelvic floor questionnaire.

GPFQ Domain Median (Range)	Chemotherapy Yes: *n* = 149; No: *n* = 68	Antihormonal Therapy Yes: *n* = 69; No: *n* = 148	Pelvic Radiotherapy Yes: *n* = 17; No: *n* = 200	Pelvic Surgery Yes: *n* = 44; No: *n* = 173
Bladder	Yes: 1.3 (0–6.9)	Yes: 1.1 (0–8.2)	Yes: 2.44 (0.44–8.22)	Yes: 1.7 (0–8.2)
	No: 1.1 (0–8.2)	No: 1.6 (0–7.8)	No: 1.3 (0–7.8)	No: 1.3 (0–7.8)
	*p* = 0.447	*p* = 0.450	*p* = 0.009	*p* = 0.177
Bowel	Yes: 2.4 (0–7.1)	Yes: 2.4 (0–7.1)	Yes: 2.7 (0.3–5.3)	Yes: 2.7 (0–5.6)
	No: 2.1 (0.0–6.8)	No: 2.4 (0–6.8)	No: 2.4 (0–7.1)	No: 2.1 (0–7.1)
	*p* = 0.354	*p* = 0.985	*p* = 0.619	*p* = 0.066
Prolapse	Yes: 0 (0–4.7)	Yes: 0.0 (0–4.7)	Yes: 0.0 (0–4.7)	Yes: 0.0 (0–6.7)
	No: 0 (0–6.7)	No: 0 (0–6.7)	No: 0 (0–6.7)	No: 0 (0–4.7)
	*p* = 0.943	*p* = 0.662	*p* = 0.559	*p* = 0.761
Sexuality	Yes: 0.5 (0–6.2)	Yes: 0.0 (0–6.2)	Yes: 0.0 (0–2.4)	Yes: 0.5 (0–4.3)
	No: 0 (0–7.1)	No: 0.5 (0–7.1)	No: 0.5 (0–7.1)	No: 0.5 (0–7.1)
	*p* = 0.120	*p* = 0.343	*p* = 0.525	*p* = 0.821
Total score	Yes: 5.2 (0.5–19.1)	Yes: 4.4 (0–19.1)	Yes: 5.8 (2.6–15.5)	Yes: 6.0 (1.4–15.5)
	No: 4.3 (0–19.6)	No: 5 (0.2–19.6)	No: 4.6 (0–19.6)	No: 4.5 (0–19.6)
	*p* = 0.264	*p* = 0.805	*p* = 0.104	*p* = 0.040

Data are presented as medians with observed minimum–maximum ranges in parentheses. Each GPFQ domain score ranges from 0 to 10, and the total score ranges from 0 to 40, with higher scores indicating greater symptom severity and pelvic floor dysfunction. For each treatment modality, the *p*-value compares patients who received the respective treatment with those who did not. Treatment groups were not mutually exclusive because patients may have received more than one treatment modality. All comparisons included the 217 patients with evaluable GPFQ data; 29 patients were excluded because no evaluable GPFQ scores were available. Comparisons were performed using the Mann–Whitney U test.

## Data Availability

The dataset and materials used in this study are available from the corresponding author upon reasonable request.

## Supplementary Materials

The following supporting information can be downloaded at: https://www.mdpi.com/article/10.3390/jcm15166218/s1, File S1: Study-specific questionnaire in English.
