# Supplementary material for: Pelvic Floor Disorders in Women with Breast Cancer and Gynaecological Cancer: Real-World Data from a Prospective Multicentre Cross-Sectional Survey
Source: J Clin Med. 2026 Aug 11;15(16):6218. doi: 10.3390/jcm15166218 (PMC13513540; doi:10.3390/jcm15166218)
Supplement: Supplementary file 1 [file jcm-15-06218-s001.zip › jcm-4425275-supplementary.pdf]

File S1: Study-specific questionnaire in English

Questionnaire

Pelvic floor dysfunction in patients with gynecological malignancies – evaluation of prevalence and treatment practice

This questionnaire is used to assess pelvic floor symptoms (prolapse symptoms, urinary and fecal incontinence, sexual dysfunction) and their management. All information will be treated anonymously and confidentially.

A - Personal Information

1. Year of birth: ___________

2. Number of vaginal deliveries: ___________

3. Number of cesarean sections: ___________

4. Number of forceps/vacuum deliveries: ___________

5. Height: ___________ Current weight: ___________

6. What is your menopausal status?

☐ Premenopausal (regular cycle)

☐ Perimenopausal (irregular cycle, beginning menopause)

☐ Postmenopausal (no menstruation for at least 12 months)

7. Which comorbidities do you have?

☐ Hypertension

☐ Diabetes type I or II

☐ Thyroid dysfunction

☐ Mental illness

☐ Other: ___________

8. Do you smoke?

☐ Yes

☐ No

9. What is your marital status?

☐ Single

☐ Married/in a partnership

☐ Divorced/separated

☐ Widowed

10. What is your highest level of education?

☐ No school qualification

☐ Secondary school certificate

☐ High school diploma

☐ Vocational training

☐ University/college degree

11. Diagnosis of your gynecological cancer:

☐ Uterine cancer

☐ Cervical cancer

☐ Ovarian cancer

☐ Vulvar/vaginal cancer

☐ Breast cancer

☐ Other: ___________

12. Year of initial diagnosis: ___________

13. Are you currently receiving cancer treatment?

☐ Yes

☐ No

14. Which cancer treatments have you ever received? (multiple answers possible)

☐ Antihormonal therapy

☐ Antihormonal therapy with CDK4/6 inhibitor

☐ Chemotherapy

☐ Platinum-based therapy

☐ Taxane

☐ Anthracycline

☐ Oxazaphosphorines

☐ Capecitabine

☐ Antibody therapy against HER2

☐ Antibody therapy against VEGF

☐ Targeted therapy with PARP inhibitor

☐ Radiotherapy

☐ Surgery

☐ Other: ___________

☐ I don’t know

15. If surgery was performed in the genital area, which one? (multiple answers possible)

☐ Simple hysterectomy

☐ Laparoscopic

☐ Open surgery

☐ Radical hysterectomy

☐ Laparoscopic

☐ Open surgery

☐ Adnexectomy

☐ Laparoscopic

☐ Open surgery

☐ Partial vulvectomy

☐ Partial vaginectomy

☐ Urethrectomy

☐ Partial bladder resection

☐ Pelvic exenteration

16. Are you currently in follow-up care?

☐ Yes

☐ No

17. Do you know the stage of your tumor? ___________

B – Pelvic Floor Symptoms

1. Do you currently have any of the following symptoms? (multiple answers possible)

☐ Pain or burning during urination

☐ Frequent urination

☐ Nocturia (more than twice per night)

☐ Urgency with incontinence

☐ Urgency without incontinence

☐ Urine leakage during coughing, sneezing, or exertion

☐ Difficulty emptying the bladder

☐ Prolapse symptoms/feeling of a foreign body in the vagina

☐ Vaginal dryness

☐ Pain during sexual intercourse

☐ Other: ___________

☐ None of the above

2. Which symptom bothers you the most and how much does it affect your daily life?

Scale: 0 (no impairment) – 10 (very severe impairment)

3. Did you have these symptoms before cancer treatment?

☐ Yes, the same

☐ Yes, but less severe

☐ No

4. When did these symptoms first occur in relation to your diagnosis?

☐ < 1 year

☐ 1–5 years

☐ > 5 years

5. Do your symptoms affect the following areas?

Daily life:

☐ Yes ☐ No

Social life:

☐ Yes ☐ No

Sexual well-being:

☐ Yes ☐ No ☐ Not sexually active

Mental health:

☐ Yes ☐ No

If yes, please describe:

6. Were you informed about the impact of your treatment on pelvic floor function before starting therapy?

☐ Yes ☐ No

7. Would you have liked more information before treatment?

☐ Yes ☐ No

C - Treatment and Support

1. Have your doctors ever actively asked about pelvic floor symptoms?

☐ Yes ☐ No

2. If yes, who asked you?

☐ Gynecologist (hospital)

☐ Gynecologist (private practice)

☐ Urologist

☐ General practitioner

☐ Physiotherapist

☐ Oncologist

☐ Radiation therapist

☐ Other: ___________

3. When were you asked?

☐ Before treatment

☐ During treatment

☐ During follow-up

☐ I don’t remember

4. Have you sought medical help yourself?

☐ Yes

☐ No

☐ No symptoms

5. If yes, from whom?

☐ Gynecologist

☐ Urologist

☐ General practitioner

☐ Physiotherapist

☐ Oncologist

☐ Radiation therapist

☐ Friends/acquaintances

☐ Internet/social media

☐ Other: ___________

6. If not, why not?

☐ Didn't know where to get help

☐ Didn't know treatment exists

☐ Don't want treatment

☐ Felt embarrassed

☐ Not taken seriously

☐ Other: ___________

7. What treatment did you receive?

☐ Medication (oral/vaginal)

☐ Physiotherapy/pelvic floor training

☐ Surgery

☐ Lifestyle advice

☐ Vaginal dilators

☐ No treatment

☐ Planned treatment

☐ Other: ___________

8. How satisfied are you with the outcome?

☐ Very satisfied

☐ Satisfied

☐ Neutral

☐ Dissatisfied

☐ Very dissatisfied

9. Have you attended a urogynecological specialist clinic?

☐ Yes (self-arranged)

☐ Yes (referred)

☐ No

D - Wishes and Suggestions

What additional information or support would you like?

Any other comments or suggestions?

Thank you for taking the time to complete this questionnaire.
